# IMMIGRATION EXPERIENCES AND COGNITIVE TRAJECTORIES AMONG OLDER CHINESE IMMIGRANTS

**DOI:** 10.1093/geroni/igac059.2476

**Published:** 2022-12-20

**Authors:** Fengyan Tang, Ke Li, Mary Rauktis, Iris Chi

**Affiliations:** University of Pittsburgh, Pittsburgh, Pennsylvania, United States; University of Pittsburgh, Pittsburgh, Pennsylvania, United States; University of Pittsburgh, Pittsburgh, Pennsylvania, United States; University of Southern California, Los Angeles, California, United States

## Abstract

Some studies have documented cognitive health among older immigrants in the United States; however, little is known about how the life-course immigration experiences were associated with cognitive trajectories among older Chinese immigrants. This study filled the research gap by identifying the patterns of cognitive change trajectory among older Chinese immigrants and examining the associations of immigration experiences (that is, age at migration, reasons for migration, acculturation, perceived discrimination, and preferred dialects) with cognitive trajectories. The sample comprised 2,075 participants from the Population Study of Chinese Elderly (PINE), who completed a battery of cognitive tests at four time points (2011-2019). Latent class growth analysis and multinomial logistic regression were utilized. Three latent classes of cognitive trajectories were identified: the low functioning with the fastest decline (LCF, 12%), the moderate functioning with a medium decline rate (MCF, 39%), and the high functioning with the slowest decline (HCF, 48%). Perceiving more discrimination reduced, while speaking Taishanese increased the odds of being in the LCF and MCF. High acculturation only distinguished MCF from HCF after controlling for the known factors of cognition such as age, education, and social engagement. This study identifies a group of older Chinese immigrants who are especially vulnerable to cognitive impairment, and indicates that the immigration-related risks for cognitive decline, such as late-life migration and lower acculturation, could be buffered by education in early life and social engagement post immigration. Practice and policy efforts are needed to increase socioeconomic and cultural opportunities for social integration among older immigrants.

